# Subjective Family Socioeconomic Status and Adolescents’ Attention: Blacks’ Diminished Returns

**DOI:** 10.3390/children7080080

**Published:** 2020-07-23

**Authors:** Shervin Assari, Shanika Boyce, Mohsen Bazargan

**Affiliations:** 1 Department of Family Medicine, Charles R Drew University of Medicine and Science, Los Angeles, CA 90059, USA; mohsenbazargan@cdrewu.edu; 2 Department of Pediatrics, Charles R Drew University of Medicine and Science, Los Angeles, CA 90059, USA; shanikaboyce@cdrewu.edu; 3 Department of Family Medicine, University of California, Los Angeles, CA 90095, USA

**Keywords:** race, ethnicity, socioeconomic status, adolescents, cognition, brain, attention

## Abstract

*Background:* Racial minorities, particularly non-Hispanic blacks (NHBs) in the US, experience weaker effects from their families’ socioeconomic status on tangible outcomes, a pattern called the Minorities’ Diminished Returns (MDRs) theory. These MDRs are frequently shown in the effects of the families’ socioeconomic status (SES) on NHB adolescents’ school performance. As a result of these MDRs, NHB adolescents from high SES families show a worse than expected school performance. The existing knowledge is, however, minimal about the role of attention in explaining the diminished returns of the families’ SES with regard to the adolescents’ outcomes. *Aim:* To investigate the racial differences in the effects of the subjective family SES on adolescents’ attention, we compared non-Hispanic white (NHW) and NHB adolescents to assess the effect of the subjective family SES on adolescents’ attention. *Methods:* This was a cross-sectional analysis that included 4188 adolescents from the Adolescent Brain Cognitive Development (ABCD) Study. The independent variable was the subjective family SES. The primary outcome was the adolescents’ attention to be measured by the stop-signal task (SST). The attention domain of the Child Behavior Checklist (CBCL) was also measured. *Results:* Overall, a high subjective family SES was associated with a higher task-based and CBCL-based attention. Race showed statistically significant interactions with subjective family SES in terms of adolescents’ attention outcomes. These interactions suggested that a high subjective family SES has smaller tangible effects on increasing the attention of NHB than NHW adolescents. *Conclusion:* The boosting effect of subjective family SES on attention is diminished for NHB rather than NHW adolescents. To minimize the racial gap in attention-related behaviors, such as school performance, we need to address the diminished returns of resources in the lives of NHB families. Not only should we equalize SES, but also increase the marginal returns of SES for racial minorities, particularly NHB families. Such efforts require public policies that empower NHB families to better leverage their SES resources and turn them into tangible outcomes. In addition, social policies should directly aim to alter the societal barriers that limit NHB families’ ability to effectively utilize their resources. Discrimination, segregation, and racism should be targets of our policy solutions.

## 1. Introduction

Compared to non-Hispanic white (NHW) adolescents, racial minority adolescents, particularly non-Hispanic black (NHB) adolescents, are at an increased risk of school dropout and poor academic achievement [1]. As academic success in the earlier stages of life is a gateway to future economic development and health later in life [2,3,4,5], it is imperative to close the racial inequalities early in life if we wish to eliminate subsequent inequalities during adulthood [2,3,4,5].

Closely associated with race is family socioeconomic status (SES) [6,7,8]. Race and SES have combined effects on adolescents’ development and health outcomes [6,7,8]. This is mainly because both low SES and racial minority status reflect marginalization, economic adversities, stress, and financial difficulties [9,10,11,12]. One of the strongest social determinants of adolescents’ developmental, behavioral, and health outcomes, is family SES [13,14,15,16]. A high family SES is linked to a wide range of positive outcomes for adolescents across domains [13,14,15,16]. Regardless of the domain, studies have established a link between low SES and the risk of behavioral and health problems among adolescents [17,18,19]. High SES families have a higher investment and involvement in the lives of their adolescents [20,21,22]. Adolescents from high SES families are also sent to better schools which have more educational resources [23,24,25]. High SES adolescents also have access to a wide range of educational and stimulating resources in their homes [26]. Finally, high SES adolescents are being raised in environments which are low in stress [27,28,29,30,31]. All these factors have strong positive effects on adolescents’ development [32,33,34,35,36].

Subjective and objective measures of SES capture different aspects of SES [37]. While education, income, employment, marital status, and wealth reflect the objective elements of SES [38], there are aspects of SES that are not reflected in objective SES measures. Subjective SES reflects aspects such as the sufficiency of economic means, financial strains, and a sense of social status relative to others. Research has shown that subjective SES may have some effects on health that are not explained by objective SES [37,39,40,41,42,43]. For racial and ethnic minorities, subjective SES may even better explain the variance of some outcome than objective SES [44,45]. This is because objective SES may lose some of its protective effect for marginalized and racialized groups [46,47]. Thus, there is a need to study subjective SES indicators as well [45,48]. This is particularly important because most of the existing research is on objective rather than subjective SES indicators [49,50,51].

There are two approaches that researchers have taken to study the joint effects of family SES and race on adolescents’ outcomes. The first approach, a more traditional one, has tried to explain the racial gap in adolescents’ outcomes by a lower family SES of racial minority families such as NHBs [52,53,54,55]. In this view, family SES mediates (explains) the effects of race on adolescents’ outcomes [56,57,58]. As such, the belief is that enhancing family SES and closing the racial differences in SES through income redistribution policies, tax policies, and empowering racial minorities to secure gain income and accumulate wealth would be the primary strategy for ending the inequalities racial adolescents are subjected to [59,60].

The second strategy, however, proposes that SES has differential effects on adolescents’ outcomes across racial groups. The Minorities’ Diminished Returns (MDRs) theory [46,61] argues that, relative to NHWs, NHB adolescents show weaker effects from their family’s SES on their tangible outcomes. This view is supported by recent evidence suggesting that family SES indicators, such as parental education [62], family income [63,64], and marital status [65], generate more desired outcomes for NHW than NHB adolescents.

It is well established by the MDR literature that the education and income of own-self [66] and parents [67,68,69] generate unequal outcomes for diverse racial groups. NHBs may differ from NHWs in their opportunities to mobilize resources, navigate systems, and secure desirable outcomes in the presence of SES resources [61,63,68,70,71,72]. As a result of these MDRs, compared to their non-HW counterparts, NHBs with a high SES show worse than expected outcomes, despite their family SES [46,61,63,64,73].

### Aims

To extend the existing knowledge on the combined effects of race and family SES on adolescents’ outcomes, we built this study on the MDRs literature and compared racial groups of adolescents for the effects of family SES on school performance. We expected weaker effects of family SES on adolescents’ attention, as a main predictor of school performance [30,74,75] for NHB than NHW adolescents.

## 2. Methods

### 2.1. Design

**Figure 1:**
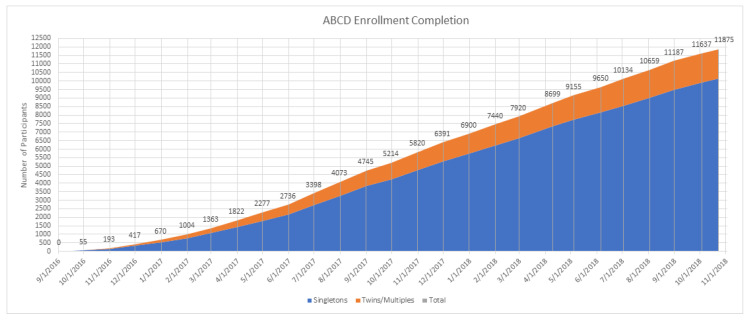
Enrolment to the Adolescent Brain Cognitive Development (ABCD) Study.

This study is a secondary analysis of wave 1 data from the Adolescent Brain Cognitive Development (ABCD) study [76,77,78,79,80], a landmark adolescents’ brain development study in the US [76,81]. The ABCD study’s baseline data were collected between 9/1/2016 and 11/1/2018 (Figure 1).

### 2.2. ABCD Sample & Sampling

In the ABCD study, participants were all adolescents aged between 9 and 10 years. The ABCD participants were recruited through a collaborative national effort that included 21 sites across various US states. The main source of recruitment in the ABCD study was schools [82]. In this analysis, we included 4188 participants. To be eligible for our analysis, the participants needed valid data on race, task-based attention, Child Behavior Checklist (CBCL)-based attention, and to be either NHB or NHW. The ABCD study sample is generalizable to the broader U.S. adolescent population [82].

### 2.3. Study Variables

Variables included race, age, sex, marital status, subjective family SES, and task-based and Child Behavior Checklist (CBCL)-based parental reports of adolescents’ attention.

#### 2.3.1. Demographic Data

Sex, age, and parental marital status were the covariate and confounders in this study. Age was reported by the parents. Sex was 1 for males and 0 for females. Parental marital status was 1 for married and 0 for any other status.

#### 2.3.2. Primary Outcome

*Adolescents’ Attention.* Attention in this study was measured using the stop-signal task (SST) [83]. In the ABCD, the SST included two runs of 180 trials. These trials showed images of a black arrows that were pointing to either the right or left. These images were displayed on a screen while the participants were in a scanner. Participants were instructed to click a button that would correspond to the direction of the arrow. They were asked to click as soon as they could see the image. They were all asked to use their dominant hand. Attention was measured as the total number of correct “Go” trials in a run. This variable was continuously a higher score, indicating a higher level of attention [84,85,86,87]. The stop-signal task is a commonly used indicator of adolescents’ attention. The SST is reliable and valid [88,89,90] and commonly used to measure attention [91,92,93]. How reliable this measure is across racial groups is still unknown.

#### 2.3.3. Secondary Outcome

*Parental Report of Adolescents’ Attention Problems.* Attention problems were measured using the Achenbach System of Empirically Based Assessment (CBCL). Attention problems are the sixth domain of the CBCL measure [94]. The attention problem scale of the CBCL measure strongly correlates with the Diagnostic and Statistical Manual of Mental Disorders (DSM-IV-TR)-based diagnosis of ADHD [95]. The CBCL instrument relied on the parental reports form to screen for social, emotional, and behavioral problems of adolescents’ behaviors and problems. The CBCL is one of the most widely used measures of adolescents’ behavioral problems including those related to poor attention, and has been used for thousands of published papers [96]. Our variable was a continuous measure. For this measure, a high score was indicative of a higher attention problem.

#### 2.3.4. Independent Variable

*Subjective Family SES.* This study measured subjective family SES using the following seven items. Participants were asked “In the past 12 months, has there been a time when you and your immediate family experienced any of the following:” (1) “Needed food but couldn’t afford to buy it or couldn’t afford to go out to get it?”, (2) “Were without telephone service because you could not afford it?”, (3) “Didn’t pay the full amount of the rent or mortgage because you could not afford it?”, (4) “Were evicted from your home for not paying the rent or mortgage?”, (5) “Had services turned off by the gas or electric company, or the oil company wouldn’t deliver oil because payments were not made?”, (6) “Had someone who needed to see a doctor or go to the hospital but didn’t go because you could not afford it?” and (7) “Had someone who needed a dentist but couldn’t go because you could not afford it?”. Responses were either 0 or 1. We calculated a mean score (a continuous measure), which ranged between 0 and 1 with a higher score, indicating a higher subjective family SES. Subjective family SES is an accepted SES indicator, as it reflects some aspects of the SES which are not captured by objective SES indicators [41,43,45,48,97,98,99]. Subjective SES is shown to have some health effects that are not seen with objective SES [37,39,40,43,45,48].

#### 2.3.5. Moderator

*Race.* Race was self-identified and was recorded as 1 for NHBs and 0 for NHWs (reference category).

### 2.4. Statistics

We used the statistical package SPSS to perform our data analysis. A Pearson bivariate test was applied to test bivariate associations. For our multivariable analysis, linear regression models were used. We ran separate models for our two outcomes: task-based attention and a CBCL-based parental report of attention problems. Results were identical for the CBCL-attention (parental report) and task-based attention. Thus, the results for models predicting parental report of attention problems are not shown (because they are identical to the models predicting task-based attention). The results are available upon request. Our first models were applied in the overall sample. Our last two models were applied in each race. *Model 1* was performed without the interaction terms. *Model 2* added an interaction term between race/ethnicity and subjective family SES. *Model 3* was performed in NHWs. *Model 4* was performed in NHBs.

### 2.5. Ethics

Our paper was exempt from a full institutional review board (IRB) review, however, the original study (ABCD) was approved by the IRB board of the University of California, San Diego (UCSD). While adolescents and parents provided assent and consent [81], given the full de-identified nature of the data, our study was non-human subject research.

## 3. Results

### 3.1. Descriptives

This study included 4188 adolescents all between 9 and 10 years old. Most participants were NHWs (*n* = 2985; 71.3%) and a minority were NHBs (*n* = 1203; 28.7%). Table 1 can be consulted for a summary of the data in the pooled sample.

Table 2 shows a correlation matrix of all the study variables in the pooled sample and by race. NHB status was associated with a lower SES and lower attention. Task-based and CBCL-based attention were positively correlated. Family SES was positively correlated with both task-based and CBCL-based attention measures.

### 3.2. Multivariate Analysis (Pooled Sample)

Table 3 shows the results of two linear regression models in the overall (pooled) sample. *Model 1* (main effect model) showed the protective effects of a high family SES on task-based attention. *Model 2* (interaction model) showed a statistically significant interaction term between race and subjective family SES on task-based attention (b = 9.33, 95% CI = 0.36 to 18.31, *p* = 0.042), suggesting that the boosting effect of a high family SES on attention is weaker for NHB adolescents relative to their NHW counterparts (Table 3).

### 3.3. Race-Stratified Models

Table 4 presents the summary of the results of two linear regression models. These models were performed in racial groups. *Model 3* showed a boosting effect of subjective family SES on the task-based attention of NHW adolescents. *Model 4* did not show any effect of a high subjective family SES on attention for NHB adolescents. While b was 11.12 for HBWs (95% CI = 4.94 to 17.31, *p* < 0.001), it was only 1.55 for NHBs (95% CI =−5.81 to 8.92, *p* = 0.679). The difference between the b corrections was statistically significant, as shown by *Model 2*.

## 4. Discussion

Overall, a high subjective family SES was associated with higher task-based and CBCL-based attention. However, the boosting effect of the subjective family SES on adolescents’ attention is diminished for NHBs than NHWs. The magnitude of the difference seems large (b was 11.12 for HBWs (95% CI = 4.94 to 17.31, *p* < 0.001), but only 1.55 for NHBs (95% CI = −5.81 to 8.92, *p* = 0.679) and was significant, statistically and clinically.

The observed diminished return of the subjective family SES on attention for the NHB compared to NHW adolescents is similar to what the previous research suggested [66,70,100,101]. MDRs are shown for various SES resources, age and developmental groups, health and behavioral outcomes, as well as types of marginalizing identities [46,61]. Across SES resources, MDRs are shown for the family income [63], education level [66], employment status [102], as well as marital status [71]. All these show that family SES results in stronger health effects for NHWs than NHBs, and this is true for adolescents [63,64,73], adults [70], and older [103] adults. Regarding the type of marginalization, MDRs hold for NHB [64], Latino [66,104,105,106] Asian American [107], Native American [108], and sexual minority [100] people. For example, Cross’ work (2020) [109] has documented racial differences in the effects of family structure on youth health. She showed that the return of living in a two-biological-parent family is weaker for NHB adolescents than NHW adolescents.

MDRs are not due to a single cause but are the result of multilevel factors and processes that operate across macro, meso, and micro levels. These may include economic, societal, psychological, and even behavioral mechanisms that carry the indirect effects of race on outcomes, across all SES levels. These include many barriers that hinder NHB people’s access to and utilization of resources. As such, MDRs reflect how racism operates even when the family is of high SES backgrounds [46,47]. Exposure to racial prejudice is higher, not lower, in the life of high SES NHB families, and interferes with the gains that are expected to follow SES resources [110,111,112]. An increased exposure [113,114,115,116,117] and vulnerability [48] to racial discrimination in high SES NHB families reduces the effects of SES, given discrimination is a risk factor for many undesired outcomes and is shown to reduce the expected gains of SES [48,116,118]. In addition, as a result of childhood poverty, adulthood SES shows a weaker effect for NHB than NHW families [119].

Due to redlining, and social stratification, residential segregation has separated the lives of NHB and NHW families. Thus, high SES NHW and NHB people are exposed to different sets of environmental and contextual risk and protective factors. As a result of such segregation, schools that high SES NHB and NHW adolescents attend are qualitatively different [120,121,122]. When high SES NHB adolescents attend poor schools, they remain at a high risk of developing educational and behavioral problems [24]. High SES NHB families may face difficulties to move out of their original neighborhoods and find new areas that are distant from the communities they used to belong to. Similarly, high SES NHB people may remain at an increased economic risk compared to high SES NHWs [69,123]. In addition, high SES NHB families remain at risk of negative environmental and toxic exposures, a pattern which is absent for NHW families with similar SES [113,114,116,124,125,126,127,128]. Similarly, high SES NHB adolescents spend time with peers with higher risk and behavioral problems than NHW adolescents with the same level of SES [62,107].

MDRs reflect a specific type of disadvantage that is not the result of low SES at an individual- or a family- level. MDRs reflect how the society fails people who have high aspirations and make it to high SES categories, because of their racial minority status. Such groups still face challenges and disadvantages regardless of their SES and middle-class status [46,61]. These MDRs are reflective of systemic racism that generates unequal outcomes despite access to equal SES resources. Combined with the low SES that affects a large part of the NHB communities, these MDRs also impact another section of the NHB population. To address social, health, and behavioral inequalities, policymakers should not take a minimalistic approach and limit their programs and plans to increase the SES of NHB communities. While SES should be improved in impoverished areas, additional policies should specifically address inequalities that are influencing the lives of NHB people across all SES and class groups [46,47].

Several scholars such as Lacy [129], Feagin and Sikes [130], and Patillo-McCoy [131], have studied the life experiences of middle-class NHB families. Reviewing the work of the above-mentioned researchers suggests that middle-class NHB families experience their social class position differently from middle-class NHW families [131]. These may be in part due to an increases in vulnerability and exposure to discrimination [48].

## 5. Conclusions

When compared to their NHW counterparts, NHB adolescents show a lower level of task-based attention and a higher level of parental-report of attention problems. NHB adolescents also have a lower level of subjective family SES. These two adversities are also compounded with a weaker association between subjective family SES and attention in NHB than NHW adolescents. As a result of the latter relative disadvantage, NHB adolescents show low attention despite across all family SES levels. It is still unknown why high-SES NHB adolescents remain at risk of undesired outcomes.

## Figures and Tables

**Table 1:** Socio-demographic data overall (*n* = 4188).

	*n*	%
**Race**		
NHWs	2985	71.3
NHBs	1203	28.7
**Sex**		
Male	2026	48.4
Female	2162	51.6
**Marital Status**		
Not Married	1323	31.6
Married	2865	68.4
	**Mean**	**SD**
**Age (Year)**	9.45	0.50
**Subjective Family SES (0–1, High)**	0.93	0.16
**Attention CBCL (0–37, Poor Attention)**	31.61	5.40
**Attention-Task (0–150, High Attention)**	122.95	22.71

CBCL = Child Behavior Checklist; SD = standard deviation; SES = socioeconomic status; NHBs = non-Hispanic blacks; NHWs = non-Hispanic whites.

**Table 2:** Correlations between the study variables **(*n* =** **4188)**.

	1	2	3	4	5	6	7
**All**							
1 Race (NHB)	1	−0.02	0.01	−0.51 **	−0.30 **	0.04 **	−0.16 **
2 Sex (Male)		1	0.03	0.00	−0.02	0.15 **	−0.01
3 Age			1	0.00	−0.02	0.00	0.11 **
4 Married				1	0.31 **	−0.11 **	0.13 **
5 Subjective Family SES (High)					1	−0.20 **	0.09 **
6 Attention Problems—CBCL (Poor)						1	−0.11 **
7 Attention—Task (Good)							1
**NHWs**							
2 Sex (Male)		1	0.03	−0.02	−0.02	0.15 **	−0.02
3 Age			1	−0.01	−0.01	0.00	0.13 **
4 Married				1	0.24 **	−0.12 **	0.05 **
5 Subjective Family SES (High)					1	−0.20 **	0.08 **
6 Attention Problems—CBCL (Poor)						1	−0.11 **
7 Attention—Task (Good)							1
**NHBs**							
2 Sex (Male)		1	0.03	0.01	−0.03	0.17 **	−0.01
3 Age			1	0.04	−0.04	−0.00	0.08 **
4 Married				1	0.16 **	−0.07 *	0.04
5 Subjective Family SES (High)					1	−0.20 **	0.02
6 Attention Problems—CBCL (Poor)						1	−0.09 **
7 Attention—Task (High)							1

* *p* < 0.05; ** *p* < 0.01. SES = socioeconomic status; NHBs = non-Hispanic blacks; NHWs = non-Hispanic whites; CBCL = Child Behavior Checklist.

**Table 3:** Summary of linear regressions overall **(*n* =** **4188)**.

	Model 1 Main Effects					Model 2 Interaction Effects				
	b	SE	95% CI		*p*	b	SE	95% CI		*p*
Race (NHBs)	−6.61	0.90	−8.37	−4.85	<0.001	−7.45	0.99	−9.38	−5.51	<0.001
Sex (Male)	−0.84	0.69	−2.19	0.51	0.223	−0.83	0.69	−2.18	0.52	0.226
Age	5.01	0.68	3.67	6.35	<0.001	5.00	0.68	3.66	6.34	<0.001
Married household	2.29	0.88	0.56	4.01	0.009	2.15	0.88	0.42	3.88	0.015
Subjective family SES (High)	−5.68	2.31	−10.21	−1.15	0.014	−11.03	3.50	−17.89	−4.18	0.002
Subjective family SES (High) × NHBs	-	-	-	-	-	9.33	4.58	0.36	18.31	0.042
Intercept	76.73	6.50	63.99	89.47	<0.001	77.17	6.50	64.43	89.91	<0.001

B = regression coefficient; CI = confidence interval; SE = standard error; SES = socioeconomic status.

**Table 4:** Linear regression models by race **(*n* =** **4188)**.

	Model 3 NHWs					Model 4 NHBs				
	b	SE	95% CI		*p*	b	SE	95% CI		*p*
Male	−0.89	0.73	−2.32	0.53	0.221	−0.69	1.58	−3.80	2.42	0.663
Age	5.31	0.72	3.89	6.73	<0.001	4.24	1.55	1.20	7.28	0.006
Married household	2.03	1.01	0.06	4.01	0.044	2.37	1.73	−1.03	5.77	0.171
Subjective family SES (High)	11.12	3.15	4.94	17.31	<0.001	1.55	3.75	−5.81	8.92	0.679
Intercept	74.35	6.92	60.78	87.92	<0.001	76.73	14.63	48.02	105.44	<0.001

B = regression coefficient; CI = confidence interval; SES = socioeconomic status SE = standard error; CI = confidence interval.
