# Erratum: Subjective Family Socioeconomic Status and Adolescents’ Attention: Blacks’ Diminished Returns. *Children* 2020, *7*, 80

**DOI:** 10.3390/children8010015

**Published:** 2020-12-31

**Authors:** Shervin Assari, Shanika Boyce, Mohsen Bazargan

**Affiliations:** 1 Department of Family Medicine, Charles R Drew University of Medicine and Science, Los Angeles, CA 90059, USA; mohsenbazargan@cdrewu.edu; 2 Department of Pediatrics, Charles R Drew University of Medicine and Science, Los Angeles, CA 90059, USA; shanikaboyce@cdrewu.edu; 3 Department of Family Medicine, University of California, Los Angeles, CA 90095, USA

The authors have requested that the following changes be made to their paper [1].

1. The following two sentences should be omitted from the Funding section of the manuscript: “Representatives from NIDA contributed to the interpretation of the data and participated in the preparation, review and approval of the manuscript. Dr. Elizabeth Hoffman was substantially involved in all of the cited grants consistent with her role as a Scientific Officer.”
2. Funding has been changed to follow as: “Shervin Assari is supported by the National Institutes of Health (NIH) grants 5S21MD000103, D084526-03, D084526-03, CA201415 02, DA035811-05, U54MD008149, U54MD007598, and U54CA229974. ABCD Funding.”
3. Conflicts of Interest has been changed to follow as: “This manuscript reflects the views of the authors and may not reflect the opinions or views of the NIH, its affiliated Institutes, Centers or offices, or ABCD consortium investigators. The authors declare no conflict of interest.”
4. Disclosure has been added as “The data used in the preparation of this article were obtained from the Adolescent Brain Cognitive Development (ABCD) study (https://abcdstudy.org) held in the NIMH Data Archive (NDA). This is a multisite, longitudinal study designed to recruit more than 10,000 children aged between 9 and 10 and follow them over 10 years into early adulthood. The ABCD study is supported by the National Institutes of Health and additional federal partners under award numbers U01DA041022, U01DA041028, U01DA041048, U01DA041089, U01DA041106, U01DA041117, U01DA041120, U01DA041134, U01DA041148, U01DA041156, U01DA041174, U24DA041123, and U24DA041147. A full list of supporters is available at https://abcdstudy.org/federal-partners.html. A listing of participating sites and a complete listing of the study investigators can be found at https://abcdstudy.org/Consortium_Members.pdf. ABCD consortium investigators designed and implemented the study and/or provided data but did not necessarily participate in the analysis or writing of this report. The ABCD data repository grows and changes over time. The ABCD data used in this report came from NIMH Data https://doi.org/10.15154/1412097.”

The authors apologize for any inconvenience caused to the readers by these changes. The changes do not affect the scientific results.
